# Effects of Combined Supplementation of *Macleaya cordata* Extract and *Benzoic Acid* on the Growth Performance, Immune Responses, Antioxidant Capacity, Intestinal Morphology, and Microbial Composition in Weaned Piglets

**DOI:** 10.3389/fvets.2021.708597

**Published:** 2021-08-18

**Authors:** Fang Wang, Yexin Yin, Mei Yang, Jiashun Chen, Chenxing Fu, Ke Huang

**Affiliations:** ^1^Animal Nutritional Genome and Germplasm Innovation Research Center, College of Animal Science and Technology, Hunan Agricultural University, Changsha, China; ^2^Hunan Collaborative Innovation Center for Utilization of Botanical Functional Ingredients, Hunan Collaborative Innovation Center of Animal Production Safety, Changsha, China

**Keywords:** *Macleaya cordata* extract, benzoic acid, immune responses, antioxidant capacity, intestine health, weaned piglets, microbial composition

## Abstract

Because the use of antibiotics is forbidden, piglets experience a considerable weanling stress, resulting in increased incidence of diarrhea and death. *Macleaya cordata* extract or benzoic acid have anti-inflammatory, antioxidant, and antimicrobial activities that makes them potential antibiotic alternatives. The objective of this study was to evaluate the potential effects of feed supplemented with *Macleaya cordata* extract and benzoic acid on growth performance, immunity, antioxidant capacity, intestinal morphology, and microflora in weaned piglets. Twenty-four weaned piglets [Duroc × (Large White × Landrace)] 28 days of age and weighing 8.41 ± 0.13 kg were randomly divided in equal numbers (*n* = 8) into three groups fed a basal diet (CON), CON + 20 mg/kg flavomycin + 50 mg/kg quinocetone (AGP), or CON + 50 mg/kg *Macleaya cordata* extract + 1,000 mg/kg benzoic acid (MB). Compared with the CON diet, dietary MB or AGP increased the final weight and average daily gain, and reduced feed efficiency and the diarrhea rate (*P* < 0.05). Compared with the CON diet, MB supplementation increased serum superoxide dismutase (SOD activity) and decreased malondialdehyde (MDA) content (*P* < 0.05). Serum interleukin (IL)-10 IgA and IgM were higher (*P* < 0.05) in MB-fed piglets than in CON-fed piglets. Piglets fed the MB diet had greater villus height and villus height to crypt depth ratio (VC) in the duodenum, villus height in the ileum, and lower crypt depth in the jejunum than did piglets given the CON diet (*P* < 0.5). Piglets in the MB group had increased concentrations of acetate, propionate, butyrate, and total short-chain fatty acids in the ileum or cecum compared with the CON and AGP groups (*P* < 0.05). *Streptococcus* proportion was lower in the MB than in the AGP group. Dietary MB increased the *Lactobacillus* and decreased *Escherichia-Shigella* populations compared with the CON group (*P* < 0.05). The study results indicate that MB can be used to replace AGP as a feed supplement for weaned piglets.

## Introduction

Weaning is one of the most stressful challenges for maintaining the growth of piglets because of unexpected changes in feeding, management, and the surrounding environment (1). The challenge may present negative effects that affect the overall condition of piglets such as immune dysfunction, change in nutritional intake and intestinal functions, as well as an increase in disease (2). Antibiotic growth promoters (AGP) are widely used as feed additives in the animal industry to promote growth and prevent disease (3). However, the inclusion of antibiotics in animal diets is a controversial issue worldwide (4). AGP was forbidden as a medicinal feed additive to promote growth following the demonstration of residues and the development of resistant strains of bacteria. Alternatives to antibiotics are currently an international research hotspot (5). Potential benefits of bioactive plant substances and organic acids for domestic animals include promoting nutrient absorption and digestion, improving animal growth performance, and promoting intestine health and immune status (6).

Sanguinarine is a naturally bioactive alkaloid obtained from *Macleaya cordata* (a perennial herb of the family *Papaveraceae*) that has antimicrobial activity, anti-inflammatory mediators, and antioxidative properties (7). Sanguinarine has been regarded as a superb animal feed additive because of its unique pharmacological characteristics and nutraceutical effects (8). Previous studies indicated that dietary supplementation of *Macleaya cordata* extract (MCE) could improve the growth performance of grass carps (9, 10), weaned pigs (11), and broilers (12). We previously reported that dietary supplementation with MCE improved the growth performance, antimicrobial activity, and intestinal development in weaned piglets (13, 14). Benzoic acid is the simplest aromatic carboxylic acid. It was approved at a dose of 0.5–1.0% in swine rearing by the European Union (15). The small intestine is the main site of benzoic acid absorption and transport by the monocarboxylic acid transporters (16). Benzoic acid supplementation has been reported to regulate the humoral immune response (17), increase antioxidant activity (18), suppress pathogens (19), promote growth performance and intestinal development (20, 21) when used as an additive in livestock nutrition. Their potential benefits make MCE or benzoic acid promising alternatives for AGP (22, 23). The available research results of these additives on the growth performance of weaned piglets are controversial, and the effect of a single additive was limited (24–27). Potential synergism of plant extracts and organic acids when used as feed supplements has been reported (28, 29), but the combined use of MCE and benzoic acid as a substitute for antibiotics has not been investigated in weaned piglets. The objective of this study was to evaluate the effects of feeding combinations of MCE and benzoic acid on the growth performance, immunity, antioxidant capacity, and intestinal morphology and microflora in weaned piglets.

## Materials and Methods

These experiments were conducted in accordance with Chinese guidelines for animal welfare and experimental protocols, and all animal procedures were approved by the Committee of Animal Care at Hunan Agricultural University (Changsha, China) (Permit Number: CACAHU 2021-00166). *Macleaya cordata* extract with more than 3.75% sanguinarine was purchased from Hunan Meikeda Biological Resources (Changsha, China). Benzoic acid with a purity of 45% and a silicon dioxide carrier were provided by Guangdong Acid Power Biotechnology Co., Ltd. (Qingyuan, China).

### Animals, Housing, and Experimental Treatments

Twenty-four weaned [Duroc × (Large White × Landrace)] 28-day-old piglets weighing 8.41 ± 0.13 kg were assigned in equal numbers (*n* = 8) to three dietary groups in a randomized complete block design according to their initial body weight. The study treatments were: (1) a corn-soybean meal basal diet (CON); (2) CON + 20 mg/kg flavomycin + 50 mg/kg quinocetone AGP; (3) CON + 50 mg/kg MCE + 1,000 mg/kg benzoic acid (MB). The pigs were individually housed in cages (150 cm long × 105 cm wide × 120 cm high) equipped with a water source. All diets were formulated to meet National Research Council (NRC, 2012) nutrient requirements (Table 1). The feeding trial lasted for 28 days. The diets and clean drinking water were provided *ad libitum*.

**Table 1:** Ingredients and chemical composition of experimental diets (as-fed basis).

**Items**	**Content (%)**
**Ingredients**	
Corn	55.00
Soybean meal	19.00
Full-fat soybean powder	10.00
Fish meal	5.00
Whey powder	6.15
Soybean oil	1.50
Dicalcium phosphate	0.90
L-Lysine-HCl	0.48
L-Threonine	0.05
DL-Methionine	0.10
L-Tryptophan	0.02
Salt	0.30
Limestone	0.50
Premix^a^	1.00
Total	100.00
**Calculated nutrients**	
Digestible energy (MJ/kg)	14.64
Crude protein	20.15
Lysine	1.38
Methionine	0.82
Methionine + cysteine	1.01
Threonine	0.97
Tryptophan	0.25
Calcium	0.80
Total phosphorus	0.73

a *The premix provided the following (per kilogram of compound feed): Vitamin A, 12,000 IU; Vitamin D, 2,500 IU; Vitamin E, 30 IU; Vitamin B12, 12 μg; Vitamin K, 3 mg; d-pantothenic acid, 15 mg; nicotinic acid, 40 mg; choline chloride, 400 mg; Mn, 40 mg; Zn, 100 mg; Fe, 90 mg; Cu, 8.8 mg; I, 0.35 mg; Se, 0.3 mg*.

### Growth Performance and Diarrhea Incidence

All pigs were weighed individually when they arrived at the experimental base. The study lasted 28 d. The individual body weight at completion of the study and the feed consumption per cage were measured and were used to calculate the average daily gain (ADG), average daily feed intake, and the feed conversion ratio (F/G), which was the feed consumption (g):weight gain (g). The diarrhea incidence and fecal consistency scores (0 = normal feces, 1 = soft feces, 2 = mild diarrhea, 3 = severe diarrhea) were determined by a trained investigator with no prior knowledge of the dietary treatment assignment. Diarrhea rate (%) was calculated as the number of pigs with diarrhea × the number of days with diarrhea/(the total number of pigs × the number of study days).

### Sample Collection and Preparation

Before the animals were euthanized by injection of pentobarbital sodium (30), blood samples were collected by anterior vena cava puncture in each treatment, centrifuged at 3,000 g at 4°C for 15 min to obtain the serum, and stored at −20°C until analysis. Segments of the mid-duodenum, mid-jejunum and mid-ileum in each animal were collected and then stored at formalin for the subsequent morphological examination. Intestinal digestive samples were collected and stored at −80°C for assays of volatile fatty acids and 16sRNA.

### DNA Extraction, Polymerase Chain Reaction Amplification, and Sequencing

Genomic DNA was extracted from the microbial community in cecal digestive samples using QIAamp Fast DNA Stool mini kits (Qiagen, Hilden, Germany) following the manufacturer's protocol. The final DNA concentration and purity were measured with NanoDrop 2000 UV-vis spectrophotometers (Thermo Scientific, Wilmington, USA); DNA quality was determined by 1% agarose gel electrophoresis. The primers for amplifying the V3–V4 hypervariable regions of the bacterial 16S rRNA genes were 338F-ACTCCTACGGGAGGCAGCAG and 806R-GGACTACHVGGGTWTCTAAT and an ABI GeneAmp^®^ 9700 PCR thermocycler (ABI, CA, USA) was used (31). The PCR amplification system and conditions have been previously described (32). The PCR products were extracted from 2% agarose gels and purified using AxyPrep DNA gel extraction kits (Axygen Biosciences, Union City, CA, USA) and quantified using a Quantus™ fluorometer (Promega, USA). All steps are performed following the manufacturers' protocols.

### Processing of Sequencing Data

The raw 16S rRNA gene sequencing reads were demultiplexed, quality filtered, and merged using previously described criteria (33) Briefly, (1) The 300 bp reads were removed from any site with an average quality score of < 20 over a 50 bp sliding window; (2) Exact barcode matching, two nucleotide mismatch, and reads with ambiguous characters were removed. Overlapping sequences exceeding 10 bp matching were assembled. The maximum mismatch ratio of the overlap region was 0.2. Reads with ambiguous characters were discarded; (3) Samples were distinguished by barcodes and primers. And the sequence direction was adjusted, with exact barcode matching and two-nucleotide mismatch in primer matching.

UPARSE version 7.0.1090 (http://www.drive5.com/uparse/) was used to cluster operational taxonomic units (OTUs) with a 97% similarity cutoff. Chimeric sequences were identified and removed (34). Mothur (version 1.30.2) (http://www.drive5.com/usearch/) was used to assess the alpha diversity of the microbiota (e.g., ACE and Chao richness estimators, Shannon and Simpson diversity indices). Beta diversity was evaluated using Principal coordinate analysis (PCoA) based on the weighted_normalized_unifrac distance using Qiime version 1.9.1 (http://qiime.org/install/index.html). Significant differences between samples were tested by analysis of similarities (ANOSIM).

### Serum Assays

Total antioxidant capacity (T-AOC), glutathione peroxidase (GSH-Px), superoxide dismutase (SOD), and malondialdehyde (MDA) concentration were assayed in serum with commercial reagent kits (Nanjing Jiancheng Bioengineering Institute, Nanjing, China). The concentrations of cytokines interleukin (IL)-1α, IL-1β, IL-2, IL-6, IL-10 and tumor necrosis factor (TNF)-α and immunoglobulin (Ig)A, IgM and IgG were assayed in serum with commercial enzyme-linked immunosorbent assay kits (Cusabio Biotechnology Co. Ltd., Wuhan, China) following the manufacturer's instructions.

### Analysis of Short Chain Fatty Acids in the Intestinal Digesta

One gram digesta of the ileal and cecal samples were weighed into a 10-mL centrifuge tube for the analysis of short chain fatty acids (SCFAs) including acetic, propionic, butyric, pentanoic, isovaleric, and isobutyric acids. After adding 6 mL of ultrapure water, the samples were homogenized and centrifuged at 12,000 × g for 15 min at 4°C. Then, 900 μL of the clear supernatant and 100 μL 25% metaphosphoric acid solution were added to a new tube and mixed. The supernatant was filtered through a 0.45-μm syringe filter and assayed by gas chromatography (Aglient 7890, Agilent Technologies, Palo Alto, CA, USA) as previously described (35).

### Intestinal Morphology

Sections of the mid-duodenum, mid-jejunum and mid-ileum in each animal were harvested and immediately fixed in 10% formalin, dehydrated in 50% ethanol, embedded paraffin, and sectioned 5 μm for hematoxylin and eosin staining. The sections were scanned and observed for histological examination by light microscopy (Nikon ECLIPSE 80i) with a computer-assisted morphometric system. The villus height, from the tip of the villi to the junction of villus and crypt, crypt depth, defined as the depth of the invagination between adjacent villi, and villus width. Ten well-oriented villi × 3 sections of each pig were used to determine these indices.

### Statistical Analysis

Experimental data was analyzed by SPSS software v. 20.0 (SPSS Inc., Chicago, IL, USA). Data were expressed as means ± standard error of the mean and significance was tested by one-way analysis of variance with a Tukey *post-hoc* test. Significant differences between mean values were defined at *P* < 0.05. The abundance of genera with significant differences between groups was assessed by the Kruskal–Wallis test.

## Results

### Growth Performance

**Figure 1:**
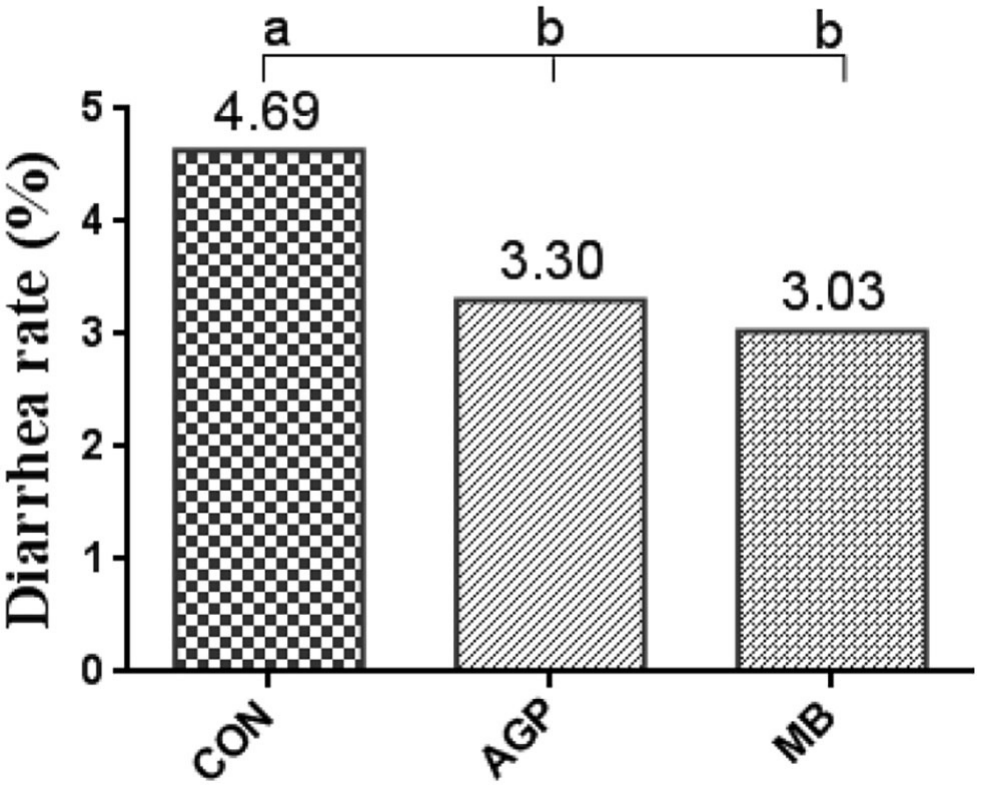
Diarrhea rate of weaned piglets fed MB dietary treatments (%) (*n* = 8). CON, basal diet; AGP, basal diet + 20 mg/kg flavomycin + 50 mg/kg quinocetone; MB, basal diet + 50 mg/kg *Macleaya cordata* extract +1,000 mg/kg benzoic acid. Different letters mean differ significantly (*P* < 0.05).

The effects of the three diets on growth performance are shown in Table 2. Compared with the CON group, dietary MB and AGP increased the final weight and decreased F/G (*P* < 0.05). However, there were no significant differences between MB and AGP treatments (*P* > 0.05). As shown in Figure 1, supplementation with MB and AGP decreased (*P* < 0.05) the diarrhea ratio by 35.00 and 28.68%, respectively, compared with piglets in the CON group.

**Table 2:** Effects of dietary *Macleaya cordata* extract and *Benzoic acid* supplementation on growth performance of weaned piglets.

**Items**	**Treatments^a^**			**SEM**	***P*-value**
	**CON**	**AGP**	**MB**		
Initial weight, kg	8.43	8.41	8.40	0.13	0.995
Final weight, kg	18.13^b^	19.48^c^	19.59^c^	0.20	0.001
ADG, g	346.22^b^	395.66^c^	399.47^c^	9.13	0.021
ADFI, g	567.34	609.25	611.45	11.80	0.236
F/G	1.64^b^	1.54^c^	1.53^c^	0.02	0.017

*SEM means standard error of the mean (n = 8). ADFI, average daily feed intake; ADG, average daily gain; F/G, Feed/gain*.

a *CON: basal diet; AGP: basal diet + 20 mg/kg flavomycin + 50 mg/kg quinocetone; MB: basal diet + 50 mg/kg Macleaya cordata extract +1,000 mg/kg benzoic acid*.

*^b, c^Different superscripts within a row indicate a significant difference (P <0.05)*.

### Effects of MB on Antioxidant Function

As shown in Table 3, compared with the CON group, supplementation with MB increased (*P* < 0.05) serum SOD activity and decreased (*P* < 0.05) the MDA content. Supplementation with AGP also decreased (*P* < 0.05) MDA content. There were no significant differences between MB treatment and AGP treatment. However, there was no difference in serum T-AOC and GSH-Px levels among the groups (P > 0.05).

**Table 3:** Effects of dietary *Macleaya cordata* extract and *Benzoic acid* supplementation on serum antioxidant activity of weaned piglets.

**Items**	**Treatments^a^**			**SEM**	***P*-value**
	**CON**	**AGP**	**MB**		
T-AOC, U/mL	3.42	3.65	3.83	0.09	0.161
GSH-Px, U/mL	311.30	318.50	337.37	7.25	0.331
SOD, U/mL	134.25^b^	137.55^bc^	145.16^c^	1.73	0.022
MDA, nmol/mL	6.50^c^	5.44^b^	5.29^b^	0.20	0.017

*SEM means standard error of the mean (n = 8). T-AOC, Total antioxidant capacity; GSH-Px, Glutathione peroxidase; SOD, Superoxide dismutase; MDA, Malondialdehyde*.

a *CON: basal diet; AGP: basal diet + 20 mg/kg flavomycin + 50 mg/kg quinocetone; MB: basal diet + 50 mg/kg Macleaya cordata extract +1,000 mg/kg benzoic acid*.

*^b, c^Different superscripts within a row indicate a significant difference (P <0.05)*.

### Effects of MB on Immune Function

The results of the effects on immune-related parameters in serum are shown in Table 4. The MB group had higher (*P* < 0.05) serum IgM and IL-10 concentrations than the CON group. The serum content of IgA was increased (*P* < 0.01) in piglets fed MB and AGP compared with those fed CON. However, differences in the levels of IgG, IL-1β, IL-2, IL-6 and TNF-α in serum in the three study groups were not significant (*P* > 0.05).

**Table 4:** Effects of dietary *Macleaya cordata* extract and *Benzoic acid* supplementation on immune property of weaned piglets.

**Items**	**Treatments^a^**			**SEM**	***P*-value**
	**Control**	**AGP**	**MB**		
IgG, g/L	7.90	8.08	9.13	0.04	0.211
IgA, g/L	1.85^b^	2.20^c^	2.19^c^	0.06	<0.01
IgM, g/L	0.96^b^	1.25^bc^	1.30^c^	0.31	0.021
IL-1α, pg/mL	52.11	57.47	61.53	2.14	0.202
IL-1β, pg/mL	43.81	52.45	61.28	4.52	0.300
IL-2, pg/mL	54.99	61.94	50.38	4.02	0.518
IL-6, pg/mL	6.45	5.20	3.04	0.80	0.218
IL-10, pg/mL	8.78^b^	10.80^bc^	12.92^c^	0.56	0.005
TNF-α, pg/mL	12.92	13.67	14.53	0.43	0.333

*SEM means standard error of the mean (n = 8). IgG, Immunoglobulin G; IgA, Immunoglobulin A; IgM, Immunoglobulin M; IL-1α, interleukin 1α; IL-1β, interleukin 1β; IL-2, interleukin 2; IL-6, interleukin 6; IL-10, interleukin 10; TNF-α, tumor necrosis factor α*.

a *CON: basal diet; AGP: basal diet + 20 mg/kg flavomycin + 50 mg/kg quinocetone; MB: basal diet + 50 mg/kg Macleaya cordata extract +1,000 mg/kg benzoic acid*.

*^b, c^Different superscripts within a row indicate a significant difference (P <0.05)*.

### Intestinal Morphology

**Figure 2:**
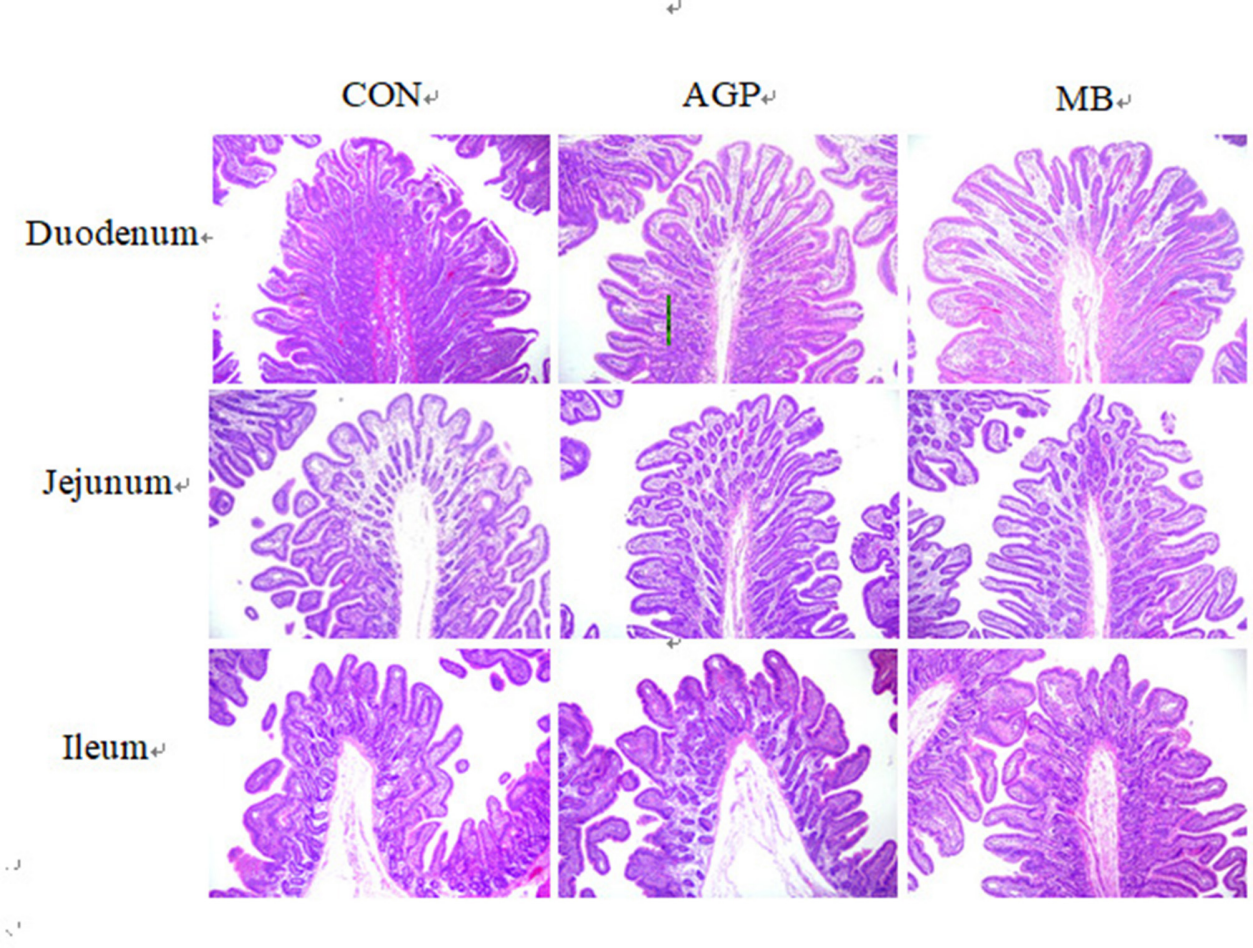
Detection of small intestine morphology of weaned piglets [H&E, 40X]. CON, basal diet; AGP, basal diet + 20 mg/kg flavomycin + 50 mg/kg quinocetone; MB, basal diet + 50 mg/kg *Macleaya cordata* extract +1,000 mg/kg benzoic acid.

The results of duodenum, jejunum, and ileum morphology are shown in Table 5 and Figure 2. Compared with the CON group, supplementation with MB increased (*P* < 0.05) villus height and the villus height-to-crypt depth ratio (VC) in the duodenum and villus height in the ileum, but reduced (*P* < 0.05) the crypt depth in the jejunum.

**Table 5:** Effects of dietary *Macleaya cordata* extract and *Benzoic acid* supplementation on intestinal morphology of weaned piglets.

**Items**	**Treatments^a^**			**SEM**	***P*-value**
	**Control**	**AGP**	**MB**		
Villus height, μm					
Duodenum	305.13^b^	320.53^bc^	332.27^c^	4.39	0.037
Jejunum	265.80	267.45	267.68	3.81	0.977
Ileum	233.68^b^	240.79^bc^	256.72^c^	3.96	0.048
Crypt depth, μm					
Duodenum	264.16	260.20	250.39	4.77	0.489
Jejunum	236.94^c^	227.59^bc^	221.70^b^	2.52	0.041
Ileum	173.13	166.03	165.54	3.81	0.670
VC, μm: μm					
Duodenum	1.16^b^	1.26^bc^	1.36^c^	0.03	0.033
Jejunum	1.13	1.19	1.21	0.02	0.225
Ileum	1.39	1.47	1.57	0.04	0.130

*SEM means standard error of the mean (n = 8). VC, the Villus height to Crypt depth rate*.

a *CON: basal diet; AGP: basal diet + 20 mg/kg flavomycin + 50 mg/kg quinocetone; MB: basal diet + 50 mg/kg Macleaya cordata extract +1,000 mg/kg benzoic acid*.

*^b, c^Different superscripts within a row indicate a significant difference (P <0.05)*.

### Microbial Composition of the Cecum Digesta

**Figure 3:**
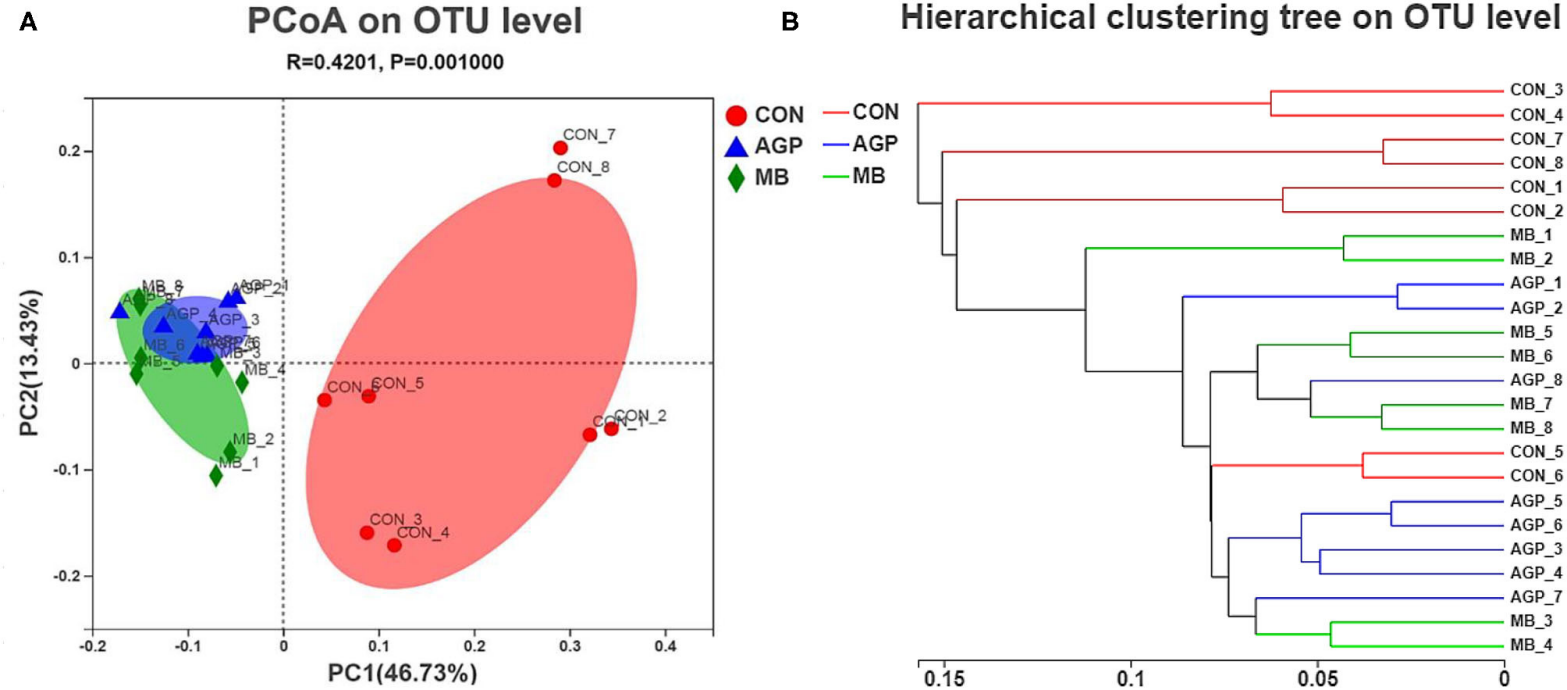
**(A)** Principl coordinates analysis (PCoA) of microbial composition in the cecal digesta of weaned piglets (based on the weighted_normalized_unifrac distance). **(B)** Analysis of hierarchical clustering tree on OTU level showed that the microbial composition of CON group had clearly distinct with the other two groups as they located distributed almost in another branches. CON, basal diet; AGP, basal diet + 20 mg/kg flavomycin + 50 mg/kg quinocetone; MB, basal diet + 50 mg/kg *Macleaya cordata* extract +1,000 mg/kg benzoic acid.

A total of 1,215,583 high-quality sequences were obtained from 24 intestinal samples. High-quality reads were clustered by > 97% sequence identity into 918 microbial OTUs. The composition of bacteria can be seen in the mean-based rarefaction curves shown in Supplementary Figure 1, in which can be found that each sample demonstrated adequate sequences. The species richness and diversity indices are shown in Supplementary Figure 2. Compared with the CON group, MB and AGP supplementation increased (*P* < 0.01) the ACE (Supplementary Figure 2A) and Chao indices (Supplementary Figure 2B). The ACE and Chao indices of the MB and AGP groups were significantly different (*P* < 0.05), but neither the Simpson (Supplementary Figure 2C) nor the Shannon (Supplementary Figure 2D) indices of the three treatment groups were significantly different. Pigs fed MB had a greater richness of intestinal microbiota compared with piglets that were fed the AGP. Principal component analysis based on the weighted_normalized_unifrac distance revealed that the bacterial community of the CON group was significantly separated from those of the AGP and MB samples (Figure 3A), indicating that the microbial composition and structure of the CON group piglets was different from those of the AGP and MB group piglets. The hierarchical clustering tree shows that the microbial composition of CON was almost entirely gathered in another branch (Figure 3B). Significant differences among the microbial composition of the study groups were revealed by ANOSIM as (*r* = 0.4201, *P* < 0. 01, among the CON, AGP, and MB groups; *r* = 0.6507, *P* < 0.01 for AGP vs. CON; *r* = 0.6468, *P* < 0.01 for MB vs. CON; and *r* = 0.0731, *P* = 0.156 for AGP vs. MB).

**Figure 4:**
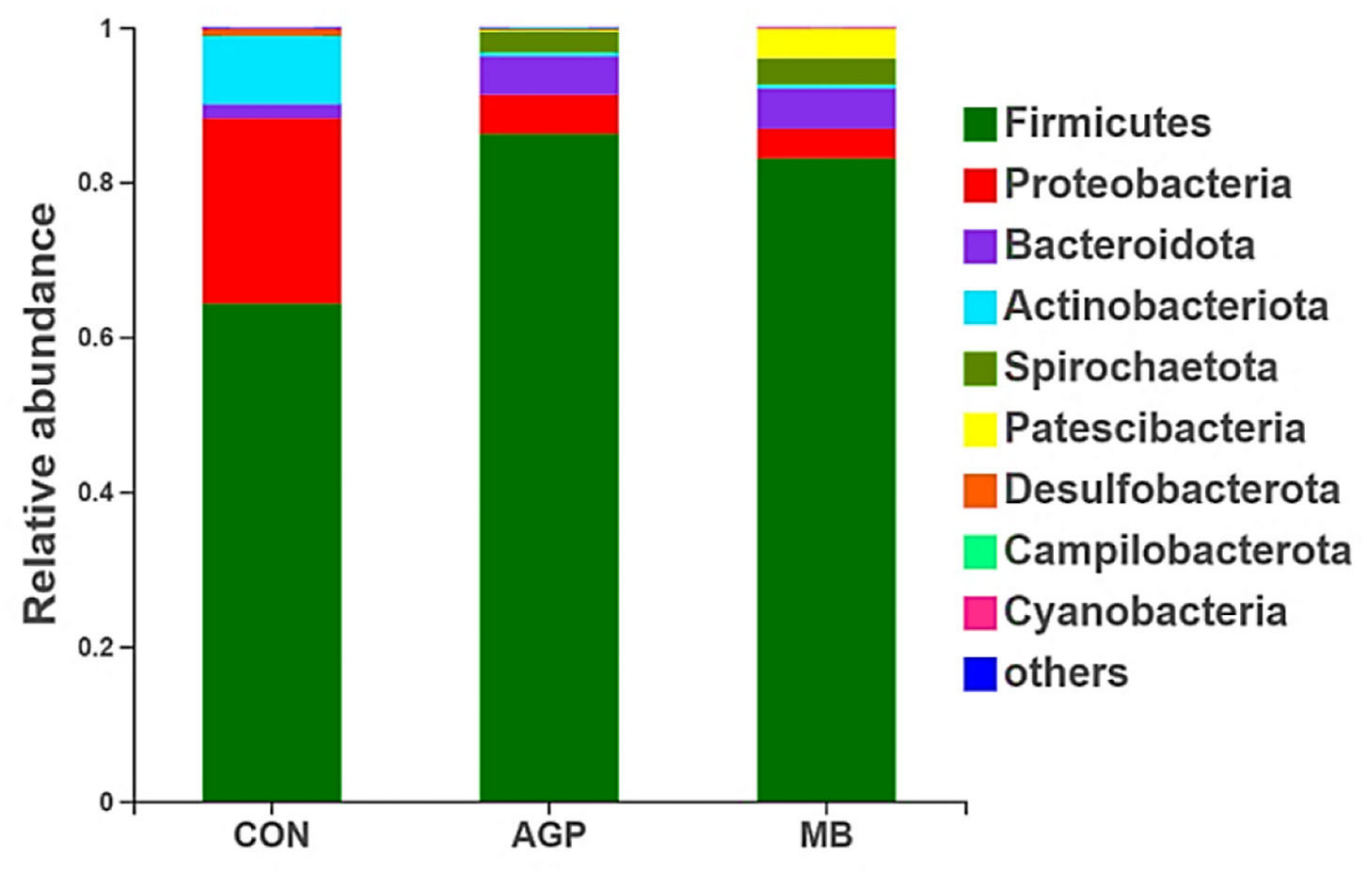
Relative abundance of intestinal bacteria based on the Phyum level. CON, basal diet; AGP, basal diet + 20 mg/kg flavomycin + 50 mg/kg quinocetone; MB, basal diet + 50 mg/kg *Macleaya cordata* extract +1,000 mg/kg benzoic acid.

Nine phyla were identified in the cecal digesta of the weaned piglets (*Firmicutes, Proteobacteria, Bacteroidota, Actinobacteriota, Spriochaetota, Patescibacteria, Desulfobacterota, Campilobacterota* and *Cyanobacteria*). Of those, *Firmicutes, Proteobacteria* and *Bacteroidota* were dominant, comprising 64.29, 23.96 and 1.77% of the CON group; 86.23, 5.10 and 4.91% of the AGP group; and 83.06, 3.82, and 2.20% of the MB group, respectively (Figure 4).

**Figure 5:**
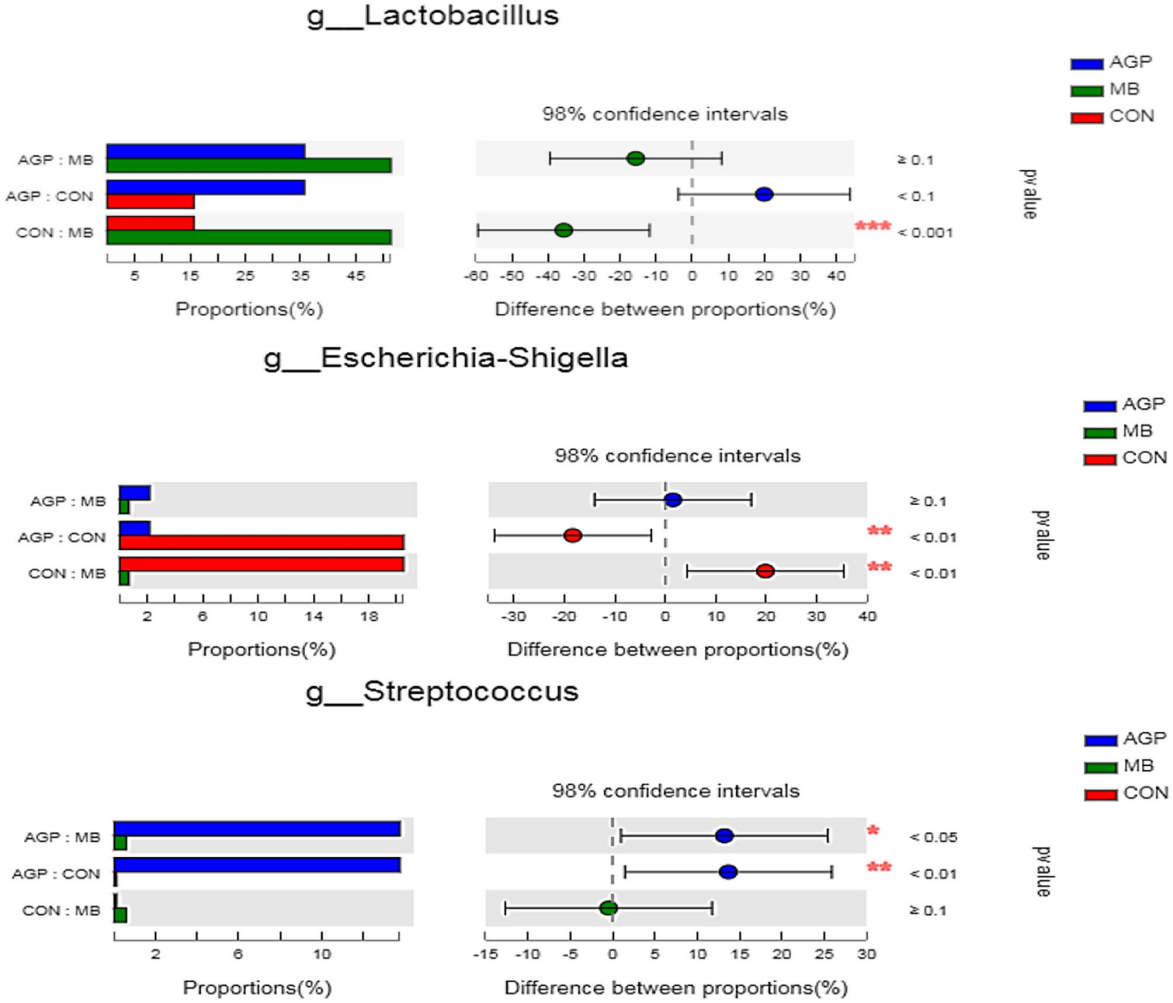
Comparative analysis of 3 most relative abundances of gut microbiota at the genera level. Kruskal–Wallis test followed by Tukey test was used to evaluate the statistical significance. Asterisks express statistical differences between different groups: *0.01 < *P* ≤ 0.05, **0.001 < *P* ≤ 0.01, ***P ≤ 0.001. CON, basal diet; AGP, basal diet + 20 mg/kg flavomycin + 50 mg/kg quinocetone; MB, basal diet + 50 mg/kg *Macleaya cordata* extract +1,000 mg/kg benzoic acid.

The 20 predominant genera of the cecal digesta are shown in a heatmap (Supplementary Figure 3). *Lactobacillus, Escherichia-Shigella* and *Streptococcus* were the three dominant genera in all samples. Compared with the CON group, dietary MB increased (*P* < 0.01) the abundance of *Lactobacillus* and decreased (*P* < 0.05) *Escherichia-Shigella* (Figure 5). Pigs supplemented with the AGP diet also had a less (*P* < 0.05) abundant *Escherichia-Shigella* population compared with pigs fed the CON diet. Compared with the AGP diet, dietary MB and CON supplementation decreased (*P* < 0.01) the abundance of *Streptococcus*.

### SCFAs

The effects of the study treatments on microbial metabolism in intestine are presented in Table 6. Piglets receiving MB had increased (*P* < 0.05) the ileal concentrations of acetate, propionate, butyrate, and total SCFAs compared with the CON and AGP groups. Compared with the CON and AGP groups, supplementation with MB increased (*P* < 0.05) the acetate, propionate, and total SCFA concentrations, with a tendency toward improvement of the butyrate concentration in the cecum (*P* = 0.093).

**Table 6:** Effects of dietary *Macleaya cordata* extract and *Benzoic acid* supplementation on short chain fatty acids in intestines of weaned piglets.

**Component (mg/g)**	**Treatments^a^**			**SEM**	***P*-value**
	**Control**	**AGP**	**MB**		
Ileum					
Acetate	0.499^b^	0.488^b^	0.583^c^	0.017	0.023
Propionate	0.097^b^	0.095^b^	0.105^c^	0.002	0.014
Butyrate	0.060^b^	0.061^b^	0.067^c^	0.001	0.026
Valerate	0.075	0.092	0.082	0.046	0.323
Isobutyrate	0.015	0.015	0.013	0.001	0.156
Isovalerate	0.013	0.010	0.009	0.001	0.401
Total SCFAs	0.759^b^	0.760^b^	0.859^c^	0.018	0.022
Cecum					
Acetate	3.297^b^	3.255^b^	3.682^c^	0.174	0.025
Propionate	2.365^b^	2.297^b^	2.872^c^	0.243	0.020
Butyrate	1.732	1.655	2.034	0.157	0.093
Valerate	0.209	0.172	0.177	0.032	0.228
Isobutyrate	0.087	0.106	0.69	0.008	0.302
Isovalerate	0.129	0.149	0.108	0.009	0.180
Total SCFAs	7.821^b^	7.632^b^	8.940^c^	0.534	*p* < 0.001

*SEM means standard error of the mean (n = 8). SCFAs, Short-chain fatty acids*.

a *CON: basal diet; AGP: basal diet + 20 mg/kg flavomycin + 50 mg/kg quinocetone; MB: basal diet + 50 mg/kg Macleaya cordata extract +1,000 mg/kg benzoic acid*.

*^b, c^Different superscripts within a row indicate a significant difference (P <0.05)*.

## Discussion

Organic acids, plant extracts, prebiotics, and enzyme preparation have been used as alternative to antibiotics as feed additives for livestock production (36, 37). In swine production, MCE or benzoic acid supplementation are effective in promoting growth performance, feed efficiency and preventing the occurrence of diarrhea at the weaning stage (15, 38). Previous studies found that MCE benefitted the growth performance of broilers (12, 39). Kantas et al. (40) reported that MCE-supplemented diets increased the body weight and ADG and reduced F/G in weaned pigs. This study, we found that MB supplementation increased the ADG and decreased the F/G and the diarrhea rate in weaned pigs. Similar results were reported by Yang et al. (41), who reported increased final body weight and ADG in weaned piglets fed diets containing essential oils and organic acids. Previous studies have revealed that MCE improved protein retention by inhibiting the decarboxylation of aromatic L-amino acids (42). Furthermore, Diao et al. (19) showed that benzoic acid could improve ADG and prevent diarrhea in weaned pigs by lowering the digesta pH and maintaining the composition of the microflora. Dietary benzoic acid has additional benefits of increasing the nutrient digestibility and improving growth performance (20–24). In conclusion, MB diet improved the growth performance of piglets, which provides new insights into the use of MB as an alternative antibiotic.

Previous studies demonstrated that weaning stress may lead to the inhibition of cellular antioxidant ability and an increase of oxidative stress (18, 43). Serum GSH-Px and SOD activities are used to evaluate non-enzymatic antioxidant defense and MDA is used as an indicators of lipid peroxidation (13). In this study, MB supplementation weaned increased serum SOD activity of SOD and decreased MDA content in weaned piglets. Previous studies found that natural antioxidants including phytochemicals, herbal plants, and vitamins exhibit antioxidant activity by scavenging reactive oxygen species (ROS) and free radicals that are present (44, 45). MCE has been shown to protect against the deterioration of physiological processes by scavenging ROS and inhibiting lipid peroxidation (12, 14, 46). Hui et al. (47) found that supplementing the diet of young pigs with 0.5% benzoic acid benzoic acid improved the activities of SOD and GSH-Px in the jejunal mucosa. Dietary supplementation with 125 mg/kg benzoic acid inhibited membrane lipid peroxidation by decreasing MDA production (48), similar results have been reported *in vitro* model (39). Data from the present study suggested that the dietary MB had a positive effect on the antioxygenic property in weaned piglets and MB did not cause oxidative stress. The available evidence suggests that dietary MB in weaned piglets may to deal with increased oxidative stress caused by weaning.

In this study, MB supplementation improved serum immunoglobulins (IgA, IgM) and IL-10 concentration in weaned pigs, which is consistent with previous studies in which supplemental 0.3 g/d MCE activated immune function and improved the growth performance of early weaned goats (38). Long et al. (49) reported that dietary supplementation with 2,000 mg/kg MCE increased the serum concentration of IgG and IgM in piglets. Serum immunoglobulins (e.g., IgG, IgA and IgM) are key indicators of immune function. Improved growth performance might be attributed to a more balanced immune steady state (50). The immune response is controlled by a complex interplay of various cytokines (11). Some proinflammatory cytokines and mediators (e.g., TNF-a, IL-6 and IL-1β) have been used as markers of anti-inflammatory activity in a dextran sodium sulfate colitis mouse model (51, 52), and IL-10 has anti-inflammatory properties and functions in immune regulation (53). Previous studies have found that dietary MCE supplementation decreased serum IL-6 and IL-1β concentrations and inhibited the progress of inflammatory disease (54). Supplementing the diet of weaned piglets with 0.8 or 1.6% benzoic acid was found to inhibit the expression of the inflammatory mediators TNF-a and IL-6 (10, 43). Furthermore, Niu et al. (44) reported that MCE supplementation increased serum IL-10 concentration. These results indicated that dietary MB may activate the immune system by reducing pro-inflammatory stimulus, and enhancing the anti-inflammatory response, suggesting that dietary MB can improve humoral immunity of pigs.

The intestine is not only a site of nutrient absorption, but also plays a crucial role in defending against external pathogens (20). A large ratio of villus height to crypt depth represents greater absorptive efficiency of the small intestine for nutrient absorption, and better resistance for disease (30, 43). However, deep crypts and short villi have been associated with increased incidence of disease (55). Halas et al. (20) reported that the addition of benzoic acid to the diet improved intestinal morphology and function in weaning pigs and then improved nutrient absorption. In this study, MB supplementation was associated with increased villus height in the duodenum and an increased V:C ratio in the duodenum compared with the CON group, both of which are indicative of improved intestinal health. Our study is consistent with previous reports that the supplementation 3.75 and 7.5 mg/kg MCE increased the V:C ratio and decreased crypt depth in the jejunum (56, 57). In addition, Lee et al. (12) found that dietary MCE supplementation resulted in a longer small intestine length, which increased nutrient utilization that was associated with improved growth performance in weaned pigs. Therefore, the improved intestinal morphology induced by MB treatment may indicate the maintenance of intestinal integrity and could contribute to an improvement in growth performance.

A balanced intestinal microbial environment plays a critical role in the development of intestinal morphology, and normal animal physiology, better control of intestinal pathogens and improved growth performance (58). In our current study, PCoA revealed that the microbial composition and structure of the CON differed from those of the AGP and MB groups, the microbial composition of weaned piglets in the MB group was similar to that of the antibiotic group. This suggests that the change in gut microbiota structure results from its accommodation to an intestinal environment created by adding MB or AGP to the diet. As described below, there were some changes in intestinal microbial composition and metabolism of cecal digesta. As weaning leads to structural changes in the small intestine and disturbance of the intestinal microbiota, resulting in the microbiota structure of the weaning changes (59). A broad pattern of dysbiosis in post-weaning diarrhea or diseases of pigs has been noted, characterized by an increase in *Proteobacteria* and other phylum (60). *Escherichia coli* and *Escherichia-Shigella* belonging to the *Proteobacteria* phylum can increase the occurrence of diarrhea (61), *Streptococcus* from the *Firmicutes* phylum can causes disease (35), while *Actinobacteriota* phylum is often found in stress-mediated environment (62). *Lactobacillus* is potentially protective of the intestine by producing antimicrobial substances that suppress the colonization of pathogenic bacteria (63). In this study, MB supplementation increased the abundance of *Lactobacillus* and decreased the abundance of harmful bacteria (i.e., *Escherichia-Shigella* and *Streptococcus*). Furthermore, *Proteobacteria* and *Actinobacteriota* in MB diet were effectively inhibited. Similarly, Diao et al. (19) reported that dietary supplementation with 5,000 mg/kg benzoic acid stimulated the growth of beneficial bacteria (i.e., *Lactobacillus* and *Bifidobacterium*) and suppressed pathogenic bacteria (i.e., *E. coli*) in weaned pigs. Bavarsadi et al. (56) reported that 3.75 and 7.50 mg/kg MCE suppressed *E. coli* and *Salmonella* counts in laying hens. *In vitro*, MCE has exhibited antimicrobial and biofilm-eliminated effects for *Streptococcus* (64). In addition, Yakhkeshi et al. (65) demonstrated that dietary supplementation with MCE modulated the intestinal microflora ecosystem by inhibiting the action of harmful bacteria and reducing the damage of intestinal epithelial cells by toxic compounds. To some extent, MB increased the intestinal beneficial bacteria population that may prevent diarrhea, keep weaned piglets healthy, and improve growth performance.

Further studies have found that dietary supplementation with MB positively modulated microbial metabolites, which may partially explain the bacteriostatic effect of MB. The intestinal digesta contains many microbial metabolites and fermentation products that reflect the status of microbial activity and intestinal health (19). The intestinal pH is associated with the proliferation of probiotics microbes, prevention of post-weaning diarrhea, and maintaining the activity of gut enzymes (21, 66). The study, results showed that dietary supplementation with MB increased the concentrations of acetate, propionate, butyrate, and total SCFAs in weaned pigs. Previous studies reported that benzoic acid regulated the production of acidic metabolites to suppress the colonization of pathogenic bacteria (67, 68). Yakhkeshi et al. (65) found that the lower pH values of the cecal in MCE-treated broiler chickens was mediated by increased SCFA concentration, which is consistent with our previous study (14). Thus, the results indicated that dietary supplementation with MB increased the SCFA concentrations and modulated microbial metabolic activity.

## Conclusions

In conclusion, dietary supplementation with MB improved the performance of weaned pigs through improvement of antioxidant capacity, immunity, and intestinal function. The moderating effect of MB on intestinal function was associated with balanced microbial composition, favorable gastrointestinal environment, and maintenance of intestinal morphology integrity. The results support the use of *Macleaya cordata* extract and benzoic acid to replace AGP based on the positive effects on performance, serum immunity, anti-oxidation activity, and intestinal health in weaned piglets.

## Data Availability Statement

The datasets presented in this study can be found in online repositories. The names of the repository/repositories and accession number(s) can be found below. The sequencing data were deposited into the Sequence Read Archive (SRA) of The National Center for Biotechnology Information (https://www.ncbi.nlm.nih.gov/sra) and can be accessed via accession number SRP320901.

## Ethics Statement

The animal study was reviewed and approved by the Committee of Animal Care at Hunan Agricultural University.

## Author Contributions

FW, JC, and CF designed the conceptualization. FW carried out formal analysis and conducted original draft preparation. FW and YY analyzed the collected data. JC, KH, and CF performed supervision. FW, YY, MY, JC, KH, and CF reviewed and edited of manuscript. All authors have read and approved to the published version of the manuscript.

## Conflict of Interest

The authors declare that the research was conducted in the absence of any commercial or financial relationships that could be construed as a potential conflict of interest.

## Publisher's Note

All claims expressed in this article are solely those of the authors and do not necessarily represent those of their affiliated organizations, or those of the publisher, the editors and the reviewers. Any product that may be evaluated in this article, or claim that may be made by its manufacturer, is not guaranteed or endorsed by the publisher.

## References

[1] Hao R Li Q Zhao J Li H Wang W Gao J. Effects of grape seed procyanidins on growth performance, immune function and antioxidant capacity in weaned piglets. Livest Sci. (2015) 178:237–42. 10.1016/j.livsci.2015.06.004

[2] Frutos J Andrés S Trevisi E Benavides J Santos N Santos A. Moderated milk replacer restriction of ewe lambs alters gut immunity parameters during the pre-weaning period and impairs liver function and animal performance during the replacement phase. Anim Feed Sci Technol. (2018) 243:80–9. 10.1016/j.anifeedsci.2018.07.009

[3] Cromwell GL. Why and how antibiotics are used in swine production. Anim Biotechnology. (2006) 13:7–27. 10.1081/ABIO-120005767 12212945

[4] Smz A Mj B Nan XB Rnm A. Antibiotics and antibiotic resistant genes (ARGs) in groundwater: a global review on dissemination, sources, interactions, environmental and human health risks. Water Res. (2020) 187:116455. 10.1016/J.WATRES.2020.116455 33032106

[5] Liu X Lv Y Xu K Xiao X Xi B Lu S. Response of ginger growth to a tetracycline-contaminated environment and residues of antibiotic and antibiotic resistance genes. Chemosphere. (2018) 201:137–43. 10.1016/j.chemosphere.2018.02.178 29524814

[6] Nguyen DH Kim IH. Protected organic acids improved growth performance, nutrient digestibility, and decreased gas emission in broilers. Animals. (2020) 10:416–29. 10.3390/ani10030416 32131472 PMC7143025

[7] Ghauri MA Su Q Ullah A Wang J Sarwar A Wu Q. Sanguinarine impedes metastasis and causes inversion of epithelial to mesenchymal transition in breast cancer. Phytomedicine. (2021) 84:153500. 10.1016/j.livsci.2007.01.063 33626427

[8] Li W Li H Yao H Mu Q Zhao G Li Y. Pharmacokinetic and anti-inflammatory effects of sanguinarine solid lipid nanoparticles. Inflammation. (2014) 37:632–8. 10.1007/s10753-013-9779-8 24272172

[9] Liu YL Zhong L Chen T Shi Y Xu SD. Dietary sanguinarine supplementation on the growth performance, immunity and intestinal health of grass carp (*Ctenopharyngodon idellus*) fed cottonseed and rapeseed meal diets. Aquaculture. (2020) 528:735521. 10.1016/j.aquaculture.2020.735521

[10] Zhang R Wang X Zhu J Liu L Liu Y Zhu H. Dietary sanguinarine affected immune response, digestive enzyme activity and intestinal microbiota of Koi carp (cryprinus carpiod). Aquaculture. (2019) 502:72–9. 10.1016/j.aquaculture.2018.12.010

[11] Li Y Li H Chu Q Xu F Liang T Zhou B. *Macleaya cordata* extracts suppressed the increase of a part of antibiotic resistance genes in fecal microorganism of weaned pigs. Can J Anim Sci. (2018) 98:884–7. 10.1139/cjas-2017-0200

[12] Lee KW Kim JS Oh ST Kang CW An BK Zhou B. Effects of dietary sanguinarine on growth performance, relative organ weight, cecal microflora, serum cholesterol level and meat quality in broiler chickens. J Poult Sci. (2015) 52:15–22. 10.2141/jpsa.0140073

[13] Chen J Kang B Yao K Fu C Zhao Y. Effects of dietary *Macleaya cordata* extract on growth performance, immune responses, antioxidant capacity, and intestinal development in weaned piglets. J Appl Anim Res. (2019) 47:349–56. 10.1080/09712119.2019.1636800

[14] Chen J Kang B Zhao Y Kang Y Fu C. Effects of natural dietary supplementation with *Macleaya cordata* extract containing sanguinarine on growth performance and gut health of early-weaned piglets. J Anim Physiol An N. (2018) 102:1666–74. 10.1111/jpn.12976 30129225

[15] Authority E. Opinion of the Scientific Panel on additives and products or substances used in animal feed (FEEDAP) on the safety and the efficacy of product “BIO-COX 120G” as feed additive in accordance with Council Directive 70/524/EEC. EFSA J. (2004) 75:1–51. 10.2903/j.efsa.2004.75

[16] Adhikari P Yadav S Cosby DE Cox NA Kim WK. Research note: effect of organic acid mixture on growth performance and Salmonella Typhimurium colonization in broiler chickens. Poult Sci. (2020) 99:2645–49. 10.1016/j.psj.2019.12.037 32359600 PMC7597380

[17] Resende M Chaves RF Garcia RM Garcia RM Barbosa JA Marques AS. Benzoic acid and essential oils modify the cecum microbiota composition in weaned piglets and improve growth performance in finishing pigs. Livest Sci. (2020) 242:104311. 10.1016/j.livsci.2020.104311

[18] Saravanan N Rajasankar S Nalini N. Antioxidant effect of 2-hydroxy-4-methoxy benzoic acid on ethanol-induced hepatotoxicity in rats. J Pharm Pharmacol. (2007) 59:445–53. 10.1211/jpp.59.3.0015 17331349

[19] Diao H Zheng P Yu B He J Mao XB Yu J. Effects of dietary supplementation with benzoic acid on intestinal morphological structure and microflora in weaned piglets. Livest Sci. (2014) 167:249–56. 10.1016/j.livsci.2014.05.029

[20] Halas D Hansen CF Hampson DJ Mullan BP Kim JC Wilson RH. Dietary supplementation with benzoic acid improves apparent ileal digestibility of total nitrogen and increases villous height and caecal microbial diversity in weaner pigs. Anim Feed Sci Technol. (2010) 160:137–47. 10.1016/j.anifeedsci.2010.07.001

[21] Guggenbuhl P Séon A Quintana A Nunes CS. Effects of dietary supplementation with benzoic acid (VevoVitall) on the zootechnical performance, the gastrointestinal microflora and the ileal digestibility of the young pig. Livest Sci. (2007) 108:218–21. 10.1016/j.livsci.2007.01.068

[22] Hu N Chen M Liu Y Shi Q Yang B Zhang H. Pharmacokinetics of sanguinarine, chelerythrine, and their metabolites in broiler chickens following oral and intravenous administration. J Vet Pharmacol Ther. (2018) 42:197–206. 10.1111/jvp.12729 30350369

[23] Kristensen NB Nørgaard JV Wamberg S Engbaek M Fernández JA Zacho HD. Absorption and metabolism of benzoic acid in growing pigs. J Anim Sci. (2009) 87:2815–22. 10.2527/jas.2009-2003 19502503

[24] Kluge H Broz J Eder K. Effect of benzoic acid on growth performance, nutrient digestibility, nitrogen balance, gastrointestinal microflora and parameters of microbial metabolism in piglets. J Anim Physiol Anim Nutr. (2006) 90:316–24. 10.1111/j.1439-0396.2005.00604.x 16867077

[25] Papadomichelakis G Mountzouris KC Zoidis E Fegeros K. Influence of dietary benzoic acid addition on nutrient digestibility and selected biochemical parameters in fattening rabbits. Anim Feed Sci Technol. (2011) 163:207–13. 10.1016/j.anifeedsci.2010.11.011

[26] Blank R Müller-Siegwardt B Wolffram S. Sanguinarine does not influence availability or metabolism of tryptophan in pigs. Livest Sci. (2010) 134:24–6. 10.1016/j.livsci.2010.06.086

[27] Bühler K Wenk C Broz J Gebert S. Influence of benzoic acid and dietary protein level on performance, nitrogen metabolism and urinary pH in growingfinishing pigs. Arch Anim Nutr. (2006) 60:382–9. 10.1080/17450390600884369 17036747

[28] Ji YJ Guo QP Yin YL Francois B Kong XF. Dietary proline supplementation alters colonic luminal microbiota and bacterial metabolite composition between days 45 and 70 of pregnancy in Huanjiang mini-pigs. J Anim Sci Biotechno. (2018) 9:370–80. 10.1186/s40104-018-0233-5 29423216 PMC5789534

[29] Liu XD Wu X Yin YL Liu YQ Geng MM Yang HS. Effects of dietary L-arginine or N-carbamylglutamate supplementation during late gestation of sows on the miR-15b/16, miR-221/222, VEGFA and eNOS expression in umbilical vein. Amino Acids. (2012) 42:2111–9. 10.1007/s00726-011-0948-5 21638020 PMC3351605

[30] Yao K Guan S Li T Huang R Wu G Ruan Z. Dietary L-arginine supplementation enhances intestinal development and expression of vascular endothelial growth factor in weanling piglets. Br J Nutr. (2011) 105:703–9. 10.1017/S000711451000365X 21219670

[31] Nan X Tan G Wang H Gai X. Effect of biochar additions to soil on nitrogen leaching, microbial biomass and bacterial community structure. Eur J Soil Biol. (2016) 74:1–8. 10.1016/j.ejsobi.2016.02.004

[32] Yang J Wang C Huang K Zhang M Pan X. Compound *Lactobacillus* sp. administration ameliorates stress and body growth through gut microbiota optimization on weaning piglets. Appl Microbiol Biot. (2020) 104:1–17. 10.1007/s00253-020-10727-4 32556411

[33] Chen S Zhou Y Chen Y Jia G. fastp: an ultra-fast all-in-one FASTQ preprocessor. Bioinformatics. (2018) 34:i884–90. 10.1093/bioinformatics/bty560 30423086 PMC6129281

[34] Stackebrandt E Goebel BM. Taxonomic note: a place for DNA-DNA reassociation and 16S rRNA sequence analysis in the present species definition in bacteriology. Int J Syst Bacteriol. (1994) 44:846–9. 10.1099/00207713-44-4-846

[35] Golomb BL Morales V Jung A Yau B Boundy-Mills Marco ML. Effects of pectinolytic yeast on the microbial composition and spoilage of olive fermentations. Food Microbiol. (2013) 33:97–106. 10.1016/j.fm.2012.09.004 23122507

[36] Chen J Kang B Qian J Han M Kang Y. Alpha-ketoglutarate in low-protein diets for growing pigs: effects on cecal microbial communities and parameters of microbial metabolism. Front Microbiol. (2018) 9:1057. 10.3389/fmicb.2018.01057 29904374 PMC5991137

[37] Fu C Guan G Wang H. The anticancer effect of sanguinarine: a review. Curr Pharm Des. (2018) 24:2760–4. 10.2174/1381612824666180829100601 30156147

[38] Yang C Cheng Y Li X Li H Yan Q He Z. Effects of dietary *Macleaya cordata* extract inclusion on transcriptomes and inflammatory response in the lower gut of early weaned goats. Anim Feed Sci Technol. (2021) 272:114792. 10.1016/j.anifeedsci.2020.114792

[39] Vieira SL Berres J Reis RN Oyarzabal OA Torres CA. Studies with sanguinarine like alkaloids as feed additive in broiler diets. Rev Bras Cienc Solo. (2008) 10:28–33. 10.1590/S1516-635X2008000100010

[40] Kantas D Papatsiros VG Tassis PD Athanasiou LV Tzika ED. The effect of a natural feed additive (*Macleaya cordata*), containing sanguinarine, on the performance and health status of weaning pigs. Anim Sci J. (2015) 86:92–8. 10.1111/asj.12240 25228334

[41] Yang C Zhang L Cao G Feng J Yue M Xu Y. Effects of dietary supplementation with essential oils and organic acids on the growth performance, immune system, fecal volatile fatty acids, and microflora community in weaned piglets. J Anim Sci. (2019) 97:133–43. 10.1093/jas/skz039 30388227 PMC6312551

[42] Drsata J Ulrichová J Walterová D. Sanguinarine and chelerythrine as inhibitors of aromatic amino acid decarboxylase. J Enzym Inhib Med Ch. (1996) 10:231–7. 10.3109/14756369609036530 8872743

[43] Pu J Chen D Gang T He J Ping Z Mao X. Protective effects of benzoic acid, bacillus coagulans, and oregano oil on intestinal injury caused by enterotoxigenic *Escherichia coli* in weaned piglets. Biomed Res Int. (2018) 2018:1–12. 10.1155/2018/1829632 30225247 PMC6129782

[44] Niu Y Wan X Zhang L Wang C He J Bai K. Effect of different doses of fermented *Ginkgo biloba* leaves on serum biochemistry, antioxidant capacity hepatic gene expression in broilers. Anim Feed Sci Technol. (2019) 248:132–40. 10.1016/j.anifeedsci.2019.01.003

[45] Jin W Fei J Xu Q Chen D He J. Alginic acid oligosaccharide accelerates weaned pig growth through regulating antioxidant capacity, immunity and intestinal development. RSC Adv. (2016) 90:870235. 10.1039/C6RA18135J

[46] Liu Y Jiao R Ma Z Liu W Wu Q Yang Z. Sanguinarine inhibits angiotensin II-induced apoptosis in H9c2 cardiac cells *via* restoring reactive oxygen species-mediated decreases in the mitochondrial membrane potential. Mol Med Rep. (2015) 12:3400–8. 10.3892/mmr.2015.3841 26017473 PMC4526052

[47] Hui D Gao Z Bing Y Zheng P He J Yu J. Effects of benzoic acid (VevoVitall) on the performance and jejunal digestive physiology in young pigs. J Anim Sci BioTechno. (2017) 7:154–60. 10.1186/s40104-016-0091-y 27239300 PMC4884408

[48] Husain K Whitworth C Trammel GL Rybak LP Somani SM. 4-Methylthiobenzoic acid protection against cisplatin nephrotoxicity: antioxidant system. Fund Appl Limnol. (1996) 32:278–84. 10.1006/faat.1996.0131 8921331

[49] Long SF Xu YT Pan L Wang QQ Wang CL Wu JY. Mixed organic acids as antibiotic substitutes improve performance, serum immunity, intestinal morphology and microbiota for weaned piglets. Anim Feed Sci Technol. (2017) 235:23–32. 10.1016/j.anifeedsci.2017.08.018

[50] Peng L Iii D. Dietary brewers yeast and the prebiotic Grobiotic AE influence growth performance, immune responses and resistance of hybrid striped bass (*Morone chrysops* × *M. saxatilis*) to *Streptococcus iniae* infection. Aquaculture. (2004) 231:445–56. 10.1016/j.aquaculture.2003.08.021 15110330

[51] Lee IA Bae EA Hyun YJ Kim DH. Dextran sulfate sodium and 2,4,6-trinitrobenzene sulfonic acid induce lipid peroxidation by the proliferation of intestinal gram-negative bacteria in mice. J Inflamm. (2010) 7:7. 10.1186/1476-9255-7-7 20181058 PMC2835697

[52] Zhao Y Yao Y Xu M Wang S Wang X Tu Y. Simulated gastrointestinal digest from preserved egg white exerts anti-inflammatory effects on Caco-2 cells and a mouse model of DSS-induced colitis. J Funct Foods. (2017) 35:655–65. 10.1016/j.jff.2017.06.028

[53] Kanai T Mikami Y Hayashi A. A breakthrough in probiotics: clostridium butyricum regulates gut homeostasis and anti-inflammatory response in inflammatory bowel disease. J Gastroenterol. (2015) 50:928–39. 10.1007/s00535-015-1084-x 25940150

[54] Niu X Fan T Li W Wei X Huang H. The anti-inflammatory effects of sanguinarine and its modulation of inflammatory mediators from peritoneal macrophages. Eur J Pharmacol. (2012) 689:262–9. 10.1016/j.ejphar.2012.05.039 22705062

[55] Kamboh AA. Flavonoids: health promoting phytochemicals for animal production-a review. J Anim Health Prod. (2015) 3:6–13. 10.14737/journal.jahp/2015/3.1.6.13

[56] Bavarsadi M Mahdavi AH Ansari-Mahyari S Jahanian E. Effects of different levels of sanguinarine on antioxidant indices, immunological responses, ileal microbial counts and jejunal morphology of laying hens fed diets with different levels of crude protein. J Anim Physiol Anim Nutr. (2016) 101:936–48. 10.1111/jpn.12528 27272257

[57] Chen JL Zheng P Zhang C Yu B He J Yu J. Benzoic acid beneficially affects growth performance of weaned pigs which was associated with changes in gut bacterial populations, morphology indices and growth factor gene expression. J Anim Physiol Anim Nutr. (2017) 101:1137–46. 10.1111/jpn.12627 27747941

[58] Chen J Yang H Long L Zhao Y Jiang Q Wu F. The effects of dietary supplementation with α-ketoglutarate on the intestinal microbiota, metabolic profiles, and ammonia levels in growing pigs. Anim Feed Sci Technol. (2017) 234:321–8. 10.1016/j.anifeedsci.2017.03.017

[59] Gart EV Welsh TH Randel RD Suchodolski JS Kintzinger J Lawhon SD. The effect of weaning stress, sex and temperament on fecal microbiota in Brahman calves. J Anim Sci. (2016) 45:22–3. 10.2527/ssasas2015-045

[60] Yang GY Zhu YH Zhang W Zhou D Zhai CC Wang JF. Infuence of orally fed a select mixture of Bacillus probiotics on intestinal T-cell migration in weaned MUC4 resistant pigs following Escherichia coli challenge. Vet Res. (2016) 47:71. 10.1186/s13567-016-0355-8 27424033 PMC4947265

[61] Alfa MJ Strang D Tappia PS Graham M Van Domselaar G Forbes JD. A randomized trial to determine the impact of a digestion resistant starch composition on the gut microbiome in older and mid-age adults. Clin Nutr. (2017) 37:797–807. 10.1016/j.clnu.2017.03.025 28410921

[62] Wen QAB He XB Shao YC Peng HA Zhao YD Pan ZAB. Impact of the gut microbiota on heat stroke rat mediated by Xuebijing metabolism. Microb Pathogenesis. (2021) 155:104861. 10.1016/j.micpath.2021.104861 33864878

[63] Yu M Mu C Zhang C Yang Y Su Y Zhu W. Marked response in microbial community and metabolism in the ileum and cecum of suckling piglets after early antibiotics exposure. Front Microbiol. (2018) 9:1166. 10.3389/fmicb.2018.01166 29899739 PMC5989621

[64] Kasuya K Yoshida E Harada R Hasegawa M. Systemic *Streptococcus* dysgalactiae subspecies equisimilis infection in a yorkshire pig with severe disseminated suppurative meningoencephalomyelitis. J Vet Med Sci. (2014) 76:715–18. 10.1292/jvms.13-0526 24419876 PMC4073340

[65] Yakhkeshi S Rahimi S Naseri KG. The Effects of comparison of Herbal extracts, antibiotic, probiotic and organic acid on serum lipids, immune response, git microbial population, intestinal morphology and performance of broilers. J Med Plants Res. (2011) 10:80–95. 10.3390/ijerph10030892 23466827 PMC3709293

[66] Beuria TK Santra MK Panda D. Sanguinarine blocks cytokinesis in bacteria by inhibiting FtsZ assembly and bundling. Biochemistry. (2005) 44:16584–93. 10.1021/bi050767 16342949

[67] Mao X Yang Q Chen D Yu B He J. Benzoic acid used as food and feed additives can regulate gut functions. Biomed Res Int. (2019) 2019:1–6. 10.1155/2019/5721585 30931328 PMC6413358

[68] Zhu L Zhao K Chen X Xu J. Impact of weaning and an antioxidant blend on intestinal barrier function and antioxidant status in pigs. J Anim Sci. (2012) 90:2581–9. 10.2527/jas.2012-4444 22896732

