# Supplementary material for: Effects of Combined Supplementation of Macleaya cordata Extract and Benzoic Acid on the Growth Performance, Immune Responses, Antioxidant Capacity, Intestinal Morphology, and Microbial Composition in Weaned Piglets
Source: Front Vet Sci. 2021 Aug 18;8:708597. doi: 10.3389/fvets.2021.708597 (PMC8416536; doi:10.3389/fvets.2021.708597)
Supplement: Supplementary file 1 [file Data_Sheet_1.pdf]

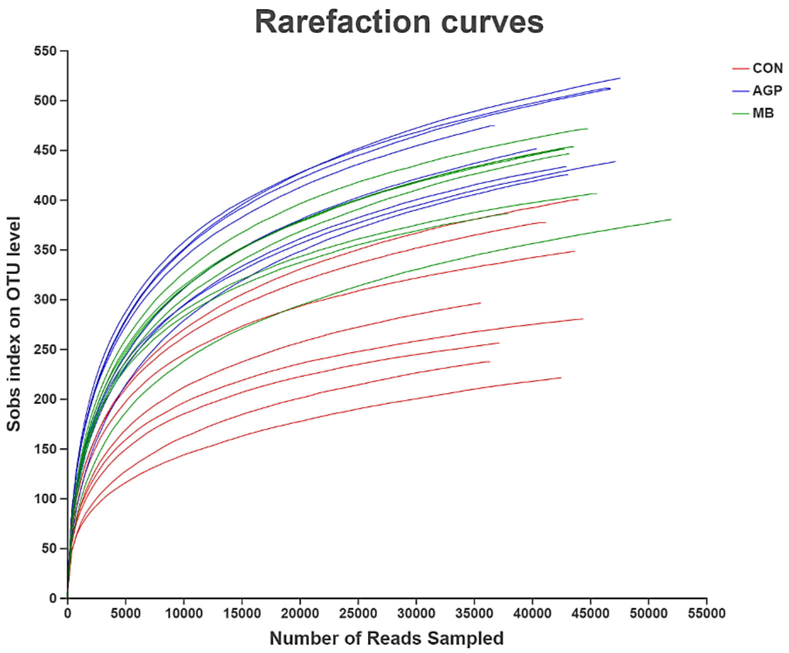

Supplementary Figure S1. Rarefaction curve for caecum digesta samples of pigs fed with CON, AGP and MB. CON: basal diet; AGP: basal diet + 20 mg/kg flavomycin + 50 mg/kg quinocetone; MB: basal diet + 50mg/kg *Macleaya cordata* extract +1000mg/kg benzoic acid.

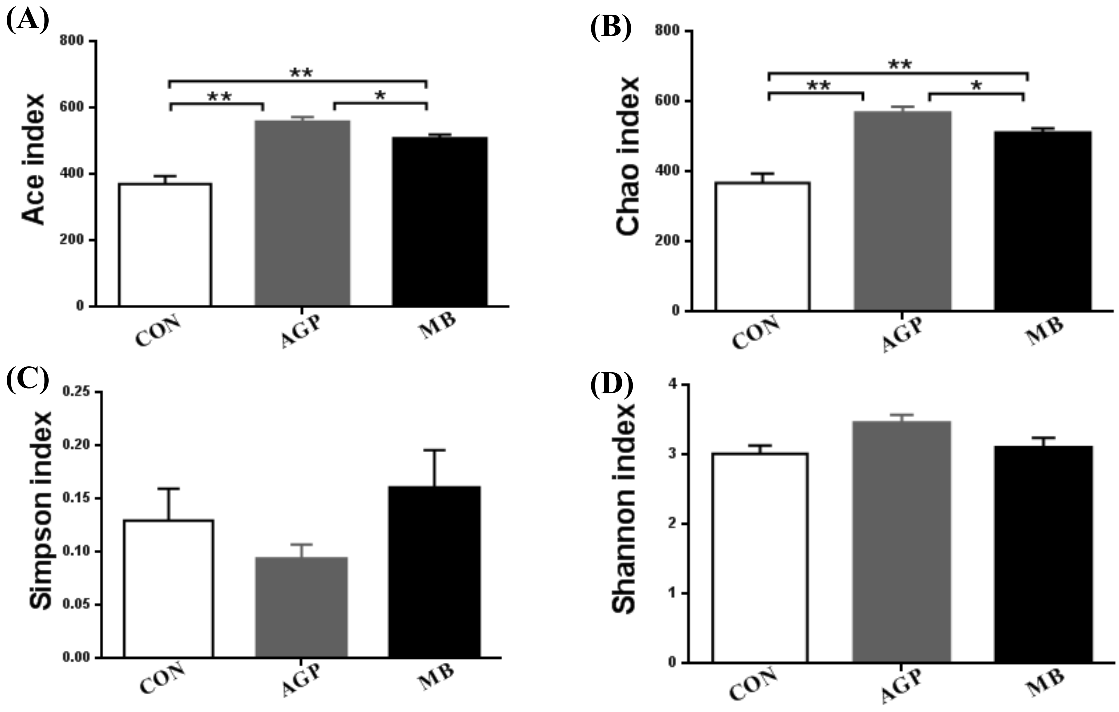

Supplementary Figure S2. The richiness and divsity index of species at 97% similarity level: Ace index (A), Chao index (B), Simpson index (C) and Shannon index (D). CON: basal diet; AGP: basal diet + 20 mg/kg flavomycin + 50 mg/kg quinocetone; MB: basal diet + 50 mg/kg *Macleaya cordata* extract +1000mg/kg benzoic acid. Asterisks indicate statistical differences between different groups: * $0.01 < P \leq 0.05$， ** $P \leq 0.01$.

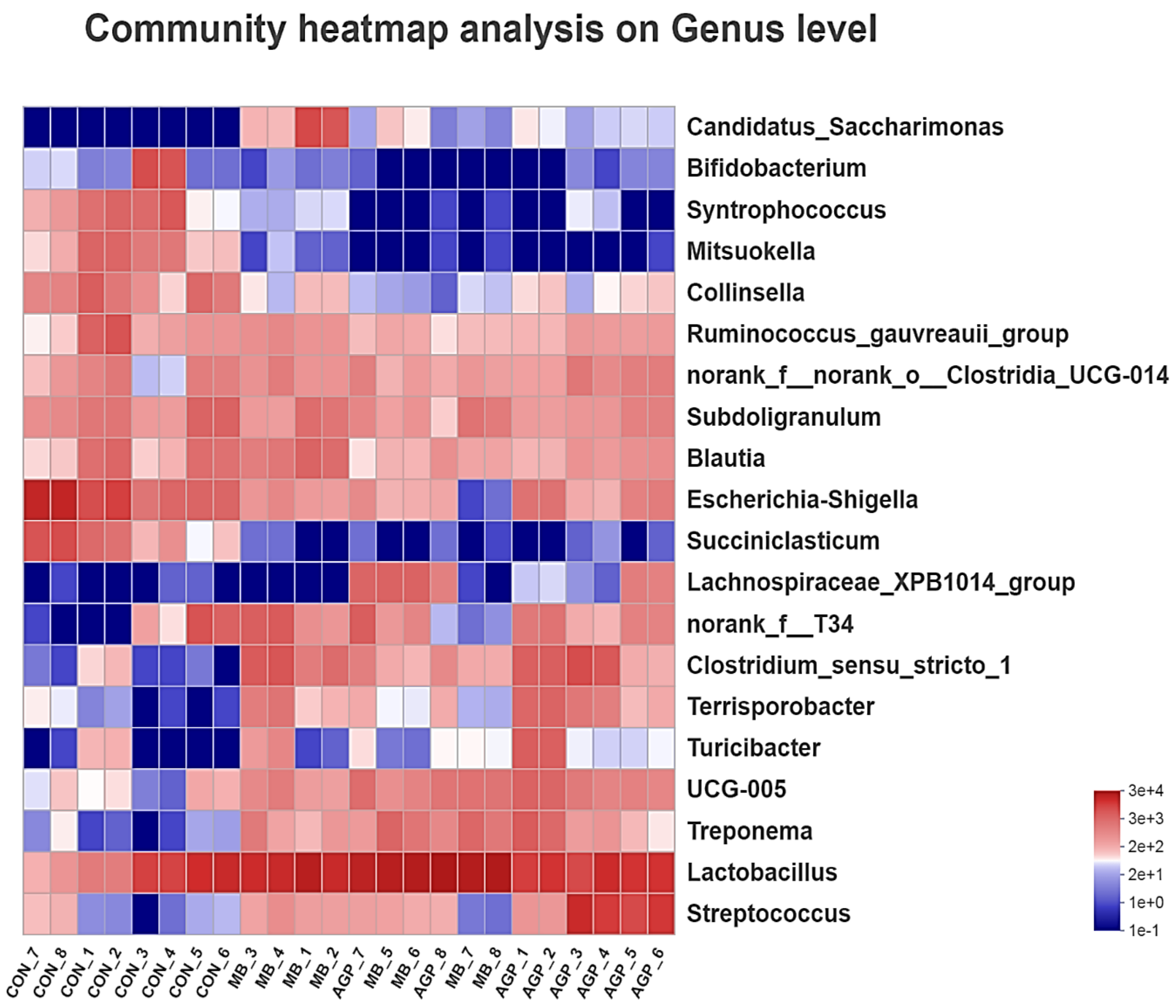

Supplementary Figure S3. Heat map based on genus for caecum digesta samples of pigs fed with CON, AGP and MB. CON: basal diet; AGP: basal diet + 20 mg/kg flavomycin + 50 mg/kg quinocetone; MB: basal diet + 50mg/kg *Macleaya cordata* extract +1000mg/kg benzoic acid.
